# Low Levels Matter: Clinical Relevance of Low Pru p 3 sIgE in Patients With Peach Allergy

**DOI:** 10.3389/falgy.2022.868267

**Published:** 2022-04-05

**Authors:** Sara Balsells-Vives, Clara San Bartolomé, Rocío Casas-Saucedo, María Ruano-Zaragoza, Josefina Rius, Maria Torradeflot, Joan Bartra, Rosa Munoz-Cano, Mariona Pascal

**Affiliations:** ^1^Clinical and Experimental Respiratory Immunoallergy (IRCE), Institut D'Investigacions Biomèdiques August Pi I Sunyer (IDIBAPS), Barcelona, Spain; ^2^Department of Immunology, Centre de Diagnòstic Biomedic (CDB), Hospital Clínic de Barcelona, Barcelona, Spain; ^3^Allergy Section, Department of Pneumology, Institut Clínic Respiratori (ICR), Hospital Clínic de Barcelona, Barcelona, Spain; ^4^Spanish Research Network on Allergy (ARADyAL, Red Nacional de Alergia–Asma, Reacciones Adversas y Alérgicas-), Instituto de Salud Carlos III, Madrid, Spain

**Keywords:** serum specific-IgE, low levels, lipid transfer protein, clinical relevance, BAT

## Abstract

Many clinical lab settings still use 0.35 KU_A_/L as the cut-off for serum specific-IgE (sIgE) immunoassays, while the detection limit is 0.1 KU_A_/L. The clinical relevance of -low-level sIgE (0.1–0.35 KU_A_/L) remains controversial. Pru p 3 sIgE is considered to be the main routine tool for assessing lipid transfer protein (LTP) sensitization. We aimed to evaluate the clinical relevance of Pru p 3 sIgE low levels in a population diagnosed with LTP allergy. Adults diagnosed with LTP allergy and Pru p 3 sIgE ≥ 0.1 KUA/L between 2012 and 2019 were included. Clinical data were reviewed. nPru p 3 basophil activation test (BAT) was performed and basophil reactivity (BR) and sensitivity (BS) correlated with the peach allergy symptoms. Pru p 3 sIgE from 496 subjects was recorded, 114 (23.0%) between 0.1 and 0.34 KU_A_/L (grLOW), the rest ≥ 0.35 KU_A_/L (grB). A total of 44.7% in grLOW and 59.9% in grB were allergic. Urticaria was more frequent in grLOW. In grLOW, Pru p 3 sIgE was higher in patients with local compared with systemic symptoms. In grB, Pru p 3 sIgE was higher in allergic patients. Pru p 3/Total IgE ratios were higher in allergic vs. tolerant in both groups. In BAT, BR was similar in both groups. In grLOW, it was higher on allergic compared with tolerant (*p* = 0.0286), and on those having systemic vs. local symptoms (*p* = 0.0286). BS showed no significant difference between groups. Patients with low levels represent a non-negligible fraction and around 45% are peach allergic. BAT showed functional sIgE in them. Pru p 3 sensitizations should be carefully evaluated even when sIgE levels are low.

## Introduction

Allergen-specific IgE (sIgE) levels cannot be used as individual predictors of clinical reactivity or severity, although high-sIgE concentrations correlate with increased risk of reactions (1). The importance of establishing sIgE cut-offs to provide clinical relevance in the assessment of food allergy has been extensively reported (2–4). The cut-off for the most common immunoassays used to quantify serum sIgE (e.g., ImmunoCAP® ThermoFisher Scientific, Uppsala), has traditionally been 0.35 KU_A_/L; and it is still used in many clinical lab settings, despite the reports showing that the cut-off may differ depending upon the factors, such as the allergenic source and patient age (3). Indeed, the technical detection limit for the *in vitro* singleplex fluorescence enzyme-immunoassay ImmunoCAP® (ThermoFisher Scientific) is 0.10 KU_A_/L. Little evidence has been reported on the clinical relevance of sIgE levels between 0.1 and 0.35 KU_A_/L and it is a matter of discussion in the field.

Lipid transfer proteins (LTPs) are widely cross-reacting panallergens related to complex clinical profiles regarding severity and food triggers (5, 6). LTPs are the most important cause of plant food allergy in adults and children in the Mediterranean, but indeed emerging in other areas (6). Pru p 3, the peach LTP, is considered to be the prototype protein, and routinely used as the main marker to assess LTPs sensitization (7). High Pru p 3 sIgE has been related with systemic reactions and a higher prevalence of hazelnut, peanut, and walnut allergy (4, 8). Pastorello et al. established Pru p 3 2.69 KU_A_/L to discriminate patients at risk of reactions (4), but other authors have found overlapped values between allergic and tolerant (9). Nevertheless, Pru p 3 allergic patients have also been reported with sIgE levels <0.35 KU_A_/L (10). We aimed to evaluate the clinical relevance of low levels of Pru p 3 sIgE by ImmunoCAP®.

## Materials and Methods

### Study Population

Adult patients evaluated in the Allergy Section of Hospital Clinic (Barcelona, Spain) between 2012 and 2019 with an LTPs food allergy and Pru p 3 sIgE ≥ 0.1 KU_A_/L were selected. Serum samples obtained following routine practice were analyzed in the Immunology Department of the same hospital. Pru p 3 sIgE (by ImmunoCAP®, Thermo Fisher Scientific) is measured per protocol in all LTPs allergic patients regardless of the presence of symptoms with peach. Sensitization to other plant food allergens was analyzed by microarray ImmunoCAP® ISAC (Thermo Fisher Scientific.) Patients sensitized to other panallergens (PR-10; TLP; Profilin) were excluded. The study was approved by the local ethic committee (HCB/2020/0373).

### Clinical Characterization

Demographical and epidemiological data were retrospectively recorded from clinical history. Peach allergy symptoms were classified as: local (gastrointestinal symptoms–GI-, Oral Allergy Syndrome–OAS-, and contact urticaria–CU-) and systemic (generalized urticaria and/or angioedema–U/AE-, anaphylaxis-AN-). Peach tolerance (–TOL-) and peach avoidance (-AV-; due to medical advice, fear, or dislike) were also recorded and also the involvement of cofactors, including exercise, alcohol, non-steroidal anti-inflammatory drugs (NSAIDs), and/or menstruation.

### Basophil Activation Test

Pru p 3 basophil activation test (BAT) was performed in some patients to assess sIgE functionality. Briefly, after the patient informed consent, 10 ml of heparinized peripheral blood was obtained and immediately taken to the laboratory for BAT using the Flow2CAST^TM^ kit (Bühlmann Laboratories AG, Switzerland) and following the manufacturer's procedures. Purified Pru p 3 (1 mg/ml, Bial Aristegui, Bilbao, Spain) was tested at 25, 12.5, 5, and 2.5 ng/ml final concentrations. Basophils were identified by flow cytometry (FACS-Canto II, BD Biosciences, Germany). A minimum of 500 basophils was gated and those CD63+ were defined as activated (≥15% was considered a positive test). Basophil reactivity (BR, i.e., number of basophils responding to a stimulus) was calculated as the CD63+ expression post-stimulus minus basal CD63+ expression, represented as % CD63+. Basophil sensitivity (BS) is calculated as CD-sens, i.e., inversion of EC50 (concentration inducing 50% of maximum response) × 100 (11).

### Statistical Analysis

Pru p 3 sIgE centralization and dispersion measurements were calculated considering a quantitative and asymmetric distribution. Free distribution was considered in our analysis so non-parametric tests were used to verify heterogenicity between our variables. Quantitative data were compared using the Mann Whitney *U*-test or the Kruskal–Wallis test. Qualitative data were compared using the chi-squared test and Fisher's exact test for a small sample size. *P* values lower than 0.05 were considered statistically significant. The GraphPad Prism 8.0.2 software (Inc., CA, USA) was used for the statistical analysis.

## Results

### Groups Characterization

A total of 496 subjects with Pru p 3 sIgE ≥ 0.1 KU_A_/L were recorded between 2012 and 2019. A total of 284 (57.3%) subjects were women, median [Interquartile range, IQR] age of 42 (17–92) years. Of 496 subjects, 114 (23.0%) had Pru p 3 sIgE between 0.1 and 0.34 KU_A_/L (grLOW = group low levels) and 382 (77.0%) ≥ 0.35 KU_A_/L (grB = group high levels).

44.7% of patients of grLOW and 59.9% in grB were allergic (*p* > 0.05), with similar peach-related symptoms and a higher presence of local symptoms. However, U/AE was more frequent in grLOW (*p* = 0.020). Peach avoidance was statistically superior in grLOW (*p* < 0.0001) (Table 1).

**Table 1:** Clinical picture.

	**grLOW *n* = 114**	**grB *n* = 328**	***P* value**
Peach allergic	44.7%	59.9%	*ns*
Peach tolerant	20.2%	25.9%	*ns*
Peach avoidance	35.1%	14.1%	^****^
**Peach-related symptoms**			
Local	50.4%	55.1%	*ns*
CU	21.9%	25.1%	*ns*
OAS	23.7%	24.6%	*ns*
GI	4.4%	5.5%	*ns*
Systemic	22.8%	25.4%	*ns*
U/AE	21.2%	17.5%	^*^
AN	1.9%	8.1%	*ns*

*Clinical relevance frequencies among studied patients. grLOW, Pru p 3 sIgE from 0.1 to 0.34 KU_A_/L; grB, Pru p 3 sIgE >0.35 KU_A_/L; CU, contact urticaria; OAS, oral allergy syndrome; GI, gastrointestinal symptoms; U/AE, generalized urticaria or angioedema; AN, anaphylaxis. Chi-squared test and Fisher's exact test for small simple size were used to test p (*

* *0.01 to 0.05*,

**** *< 0.0001 and ns, non-significant). Patients avoiding peach were not included on the symptom statistical analysis because tolerance or allergy could not be guaranteed*.

### Pru p 3 sIgE Levels and Symptoms

**Figure 1:**
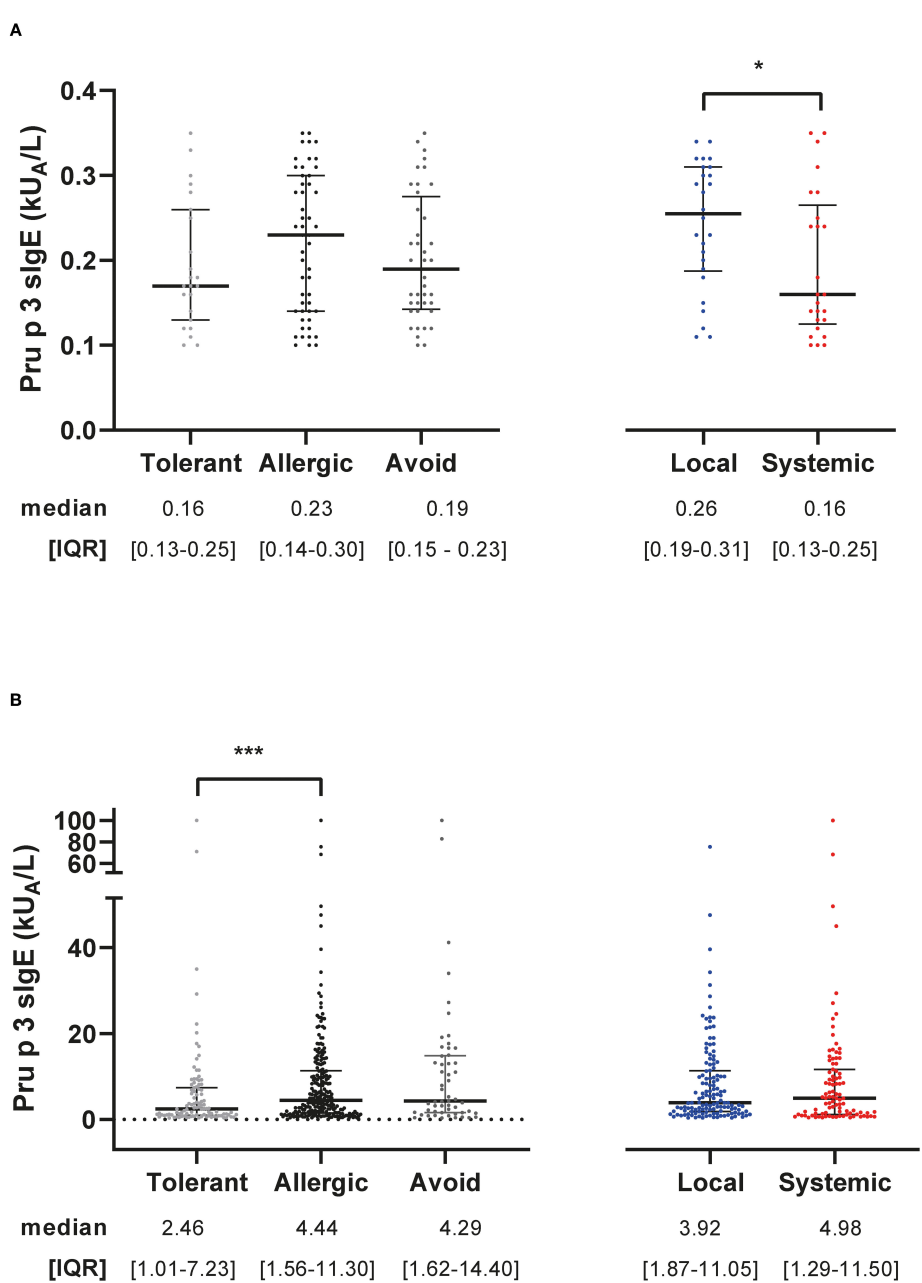
Levels of Pru p 3 sIgE per group. Pru p 3 sIgE distribution, median, and IQR (interquartile range) values from grLOW **(A)** and grB **(B)** according to symptom classification (tolerance vs. allergy, local vs. systemic). The Mann–Whitney test was used to test *p* (*0.01 to 0.05, ***0.0001 to 0.001).

Peach sIgE values were higher in grB, as well as Pru p 3/total IgE ratio (*p* < 0.05), whereas no differences were observed in Pru p 3/Peach sIgE (ratio) between groups (Table 2). In grLOW (Figure 1A), Pru p 3 sIgE was higher in patients with local compared to systemic symptoms (*p* = 0.0385). In grB (Figure 1B), Pru p 3 sIgE was higher in allergic compared to tolerant (*p* = 0.0009). The medians from the ratios Pru p 3/peach sIgE were superior to 1 for either grLOW or grB. Moreover, when classifying patients according to their clinical symptoms, no statistically significant differences were found. Pru p 3/Total IgE ratios were lower than 1% in grLOW, unlike grB. In both groups, these ratios were statistically higher (*p* < 0.0001) in allergic compared to tolerant (Supplementary Table 1).

**Table 2:** Pru p 3 sIgE values distribution.

**classification**	**Peach sIgE median [IQR] KU_**A**_/L**	**Pru p 3 sIgE median [IQR] KU_**A**_/L**	**Pru p 3/Peach sIgE median [IQR]**	**Pru p 3/Total sIgE median [IQR]**	**Pru p 3 sIgE on CCD+ median [IQR] KU_**A**_/L**
grLOW [0.1–0.35]	0.20 [0.14–0.28]	0.19 [0.07–0.26]	1.16 [0.92–1.46]	0.00 [0.00–0.01]	0.29 [0.22–0.31] *ns*
grB [≥0.35]	3.73 [1.35–10.28]	3.37 [1.16–9.67]	1.19 [1.04–1.38]	0.03 [0.01–0.07]	16.30 [4.58–20.85] p^*^
	p^***^	p^***^	*ns*	p^***^	

*IgE values distribution among groups: grLOW (Pru p 3 sIgE from 0.1 to 0.34 KU_A_/L) and grB (Pru p 3 sIgE >0.35 KU_A_/L). Pru p 3, peach and Pru p 3/Peach ratio sIgE median and IQR (interquartile range) results are included as well as CCD+ Pru p 3 sIgE median [IQR]. Differences between grLOW/grB and between CCD+/CCD- Pru p 3 sIgE in grLOW/grB were statistically evaluated with the Mann–Whitney–test (*

* *0.01 to 0.05*,

*** *0.0001 to 0.001, ns, non-significant)*.

### Co-sensitization

Co-sensitization to other LTPs was analyzed in 70 patients of grLOW and 318 of grB (Supplementary Figure 1; Supplementary Table 2). In grLOW, co-sensitization was statistically less frequent (64.3 vs. 95.9%). Mal d 3, Ara h 9, and Jug r 3 were the most frequent ones, and Tri a 14 the rarest in both groups.

Cross-reactive carbohydrate determinants (CCD) reactive sIgE may cause false-positive results in Pru p 3 measurements by binding the test cellulose matrix (12). CCD sensitization data were available for 80 (70.2%) patients of grLOW and 226 (59.2%) of grB. In grLOW, of the 7 CCD+ (8.7%), 5 avoided eating peach, 1 tolerated and 1 referred local symptoms. In grB, of the 19 CCD+ (8.4%), four avoided the ingestion of peach, three tolerated, six had local, and six systemic symptoms (two anaphylaxis). Tolerant and allergic frequencies were not statistically different between CCD+ and negative (Table 2). In grB, were found significant differences on sIgE to Pru p 3 from CCD+ compared with CCD–, being higher on CCD+.

### Basophil Activation Test Results

nPru p 3 BAT was performed on 12 patients per group as previously reported (10). All in grB were BAT+, being 3 (25%) tolerant and 9 (75%) allergic (5 local/4 systemic reactions). In grLOW (Table 3), 7 (58.3%) were BAT+: 6 (85.7%) allergic (2 local/4 systemic reactions) and 1 (14.3%) avoided peach. In BAT-: 2 (40%) were tolerant and 3 (60%) allergic (2 local/1 systemic reactions). The median [IQR] for Pru p 3 sIgE for grLOW was 0.26 [0.10–0.28] KU_A_/L. The ratio Pru p 3/peach sIgE median was 0.99 [0.79–1.09]. In addition, from these BAT- patients were 0.21 [0.18–0.23] (Pru p 3 sIgE) and 0.98 [0.97–0.99] (Pru p 3/peach sIgE ratio). BAT reactivity (BR, %CD63+ basophils) was not statistically different between groups (BR median: 17.8% grLOW/ 27.3% grB), neither when only allergic patients of each group were compared. In grLOW, BR was significantly higher on allergic individuals vs. tolerant ones (*p* = 0.0286), and on those having systemic symptoms vs. local (*p* = 0.0286). No statistically significant differences in basophil sensitivity were found between groups, although being higher in grLOW (CD-sens median: 819.0 grLOW/ 75.4 grB).

**Table 3:** Characteristics and BAT results of the allergic patients from grLOW.

	**BAT**	**EC50**	**% CD63+**				**Symptoms**	**Pru p 3 sIgE (KU_**A**_/L)**	**Ratio Pru p 3/peach sIgE**
			**2.5**	**5**	**12.5**	**25**			
			**ng/mL Pru p 3**						
P1	-	3.29	0.60	0.40	0.20	1.40	TOL	0.26	NA
P2	-	3.71	0.60	0.50	0.00	0.20	TOL	0.20	20
P3	-	32.48	0.20	0.00	0.00	0.00	CU, OAS	0.25	0.96
P4	-	-	0.00	0.00	0.00	0.00	OAS	0.30	0.73
P5	+	0.04	16.60	26.80	31.90	37.20	OAS	0.34	0.97
P6	+	0.13	16.70	2.40	14.80	0.20	GID	0.22	NA
P7	-	-	0.00	0.00	0.00	0.00	AN	0.16	1.00
P8	+	0.02	41.70	25.70	16.90	84.30	U/AE	0.12	0.52
P9	+	0.00	57.80	66.20	62.40	55.90	U/AE (exercise)	0.12	1.09
P10	+	0.09	9.20	17.40	19.60	25.20	U/AE	0.26	1.18
P11	+	0.00	54.10	48.40	59.40	60.40	SHOCK	0.29	1.07
P12	+	0.22	12.80	15.70	12.10	0.70	AVOID	0.28	0.43

*Characteristics and BAT results of the grLOW patients (n = 12) tested under a Pru p 3 stimulation. %CD63+, % of activated basophils; EC50, the concentration inducing 50% of maximum response. Tolerance (TOL), local (CU, contact urticaria; OAS, oral allergy syndrome; GI, gastrointestinal symptoms) and systemic symptoms (U/AE, generalized urticaria or angioedema; AN, anaphylaxis). In parentheses the presence of cofactors is detailed. Pru p 3 and Pru p 3/peach sIgE are included. A ratio ≥1 indicates a greater proportion of sIgE Pru p 3 compared to peach sIgE. NA, not available*.

## Conclusion

In summary, the ratio Pru p 3/Peach was similar in both groups and superior to 1, which would confirm a sensitization due to Pru p 3 on our population (13). About 45% of our patients of grLOW are allergic, highlighting the importance of considering Pru p 3 sIgE > 0.1 as potentially clinically relevant, despite 0.35 has traditionally been used as the cut-off, BAT reactivity (similar in both groups) demonstrated the presence of functional sIgE in patients with low levels.

Besides the theory reported by Kleine-Tebbe and Jakob (14) exposing that a 0.01 or greater ratio of specific IgE to total IgE, translated as a fraction of 1% of bound total IgE, is enough for basophil half-maximal activation, we observe basophil activation with a lower percentage. Thus, reliable quantitative detection of sIgE and the ratios analysis of specific and total IgE on these patients is relevant for an accurate diagnosis (13, 15).

A definite answer for the reason why Pru p 3 sIgE levels are higher on patients with local symptoms compared with those with systemic is not clear. Little is known about the real correlation between LTP sIgE levels and symptoms severity, and conflicting results have been published (9, 16, 17). It has been reported that high Pru p 3 sIgE concentrations correlate with an increased risk of reactions (18). Ciprandi et al. (19) described Pru p 3 sIgE levels variation as an age-dependent event. They reported an increase from infancy to young adulthood (highest from 21 to 30 years) that posteriorly decreased. Also, values have been inversely related with an early onset peach allergy (16).

Moreover, it has been described that mono-sensitization to LTP correlates with a more severe clinical reactivity (20) which could be explained by the fact that IgE receptors are mostly occupied by LTP sIgE, which would induce a more efficient cross-linking of the FcεRI and effector cell activation, but not actually related to sIgE levels.

In the previous studies from our group and collaborators (21–23), a trend to lower levels of sIgE has been observed in those groups with severe symptoms compared with those with mild symptoms. From our point of view, we think that this might be explained by the differential affinity of sIgE to the antigen and differential efficiency on the cross-linking in effector cells in which the ratio of sIgE to total IgE of 0.01 is enough for half-maximal activation of the effector cells.

CCD sensitization was similarly distributed in both the groups, ruling out that low levels detected were merely artifacts of CCD interaction not deserving clinical consideration.

Finally, a lower co-sensitization to other LTPs was found on grLOW although sensitization profiles (peanut, walnut, and apple) were similar in both the groups. This study has some limitations, besides being retrospective. Mainly, oral food challenges could not be done to confirm food diagnosis due to logistic limitations; and the fact that avoidance may have caused sIgE concentrations to decrease in patients with a history of a severe reaction.

In conclusion, our data show that, regardless of patients with low Pru p 3 sIgE may represent a minority in our daily practice, this sensitization can be clinically relevant, with up to 20% of systemic reactions. Therefore, Pru p 3 sensitizations should be carefully evaluated even when sIgE levels are low.

## Data Availability Statement

The original contributions presented in the study are included in the article/Supplementary Material, further inquiries can be directed to the corresponding author/s.

## Ethics Statement

The studies involving human participants were reviewed and approved by Hospital Clínic de Barcelona Ethical Committee. The patients/participants provided their written informed consent to participate in this study.

## Author Contributions

SB-V has contributed to the acquisition, analysis, and interpretation of data, as well as drafting the manuscript for publication. RC-S and MR-Z have contributed to the acquisition of clinical data. CS, JR, and MT contributed to the performance of laboratory tests. JB, RM, and MP have contributed to the design and interpretation of the data and critically revised. All authors have participated sufficiently in the work, approved the final version, agreed to be accountable for all aspects of the work in ensuring that questions related to the accuracy or integrity of any part of the work are appropriately investigated and resolved.

## Conflict of Interest

The authors declare that the research was conducted in the absence of any commercial or financial relationships that could be construed as a potential conflict of interest.

## Publisher's Note

All claims expressed in this article are solely those of the authors and do not necessarily represent those of their affiliated organizations, or those of the publisher, the editors and the reviewers. Any product that may be evaluated in this article, or claim that may be made by its manufacturer, is not guaranteed or endorsed by the publisher.
