# Supplementary material for: Low Levels Matter: Clinical Relevance of Low Pru p 3 sIgE in Patients With Peach Allergy
Source: Front Allergy. 2022 Apr 5;3:868267. doi: 10.3389/falgy.2022.868267 (PMC9234939; doi:10.3389/falgy.2022.868267)
Supplement: Supplementary file 1 [file Data_Sheet_1.docx]

Supplementary Material

# Supplementary Tables

**Supplementary Table 1. Pru p 3/Peach sIgE and Pru p 3/Total IgE ratio distribution.**

|  | Pru p 3/Peach sIgE  median [IQR]  kU_A_/L | |  | Pru p 3/Total sIgE  median [IQR] (%)  kU_A_/L | | | |
| --- | --- | --- | --- | --- | --- | --- | --- |
|  | **GrLOW** | **GrB** |  | **GrLOW** | | **GrB** | |
| Allergic | 1.13 [0.88-1.37] | 1.19 [1.04-1.36] |  | 0.004 [0.001-0.009] | (0.4%) | 0.034 [0.014-0.071] | (3.4%) |
| Tolerant | 1.27 [0.77-2.18] | 1.29 [1.02-1.60] |  | 0.001 [0.001-0.007] | (0.1%) | 0.011 [0.004-0.037] | (1.1%) |
| Avoid | 1.38 [1.18-1.79] | 1.16 [1.05-1.16] |  | 0.001 [0.000-0.002] | (0.1%) | 0.040 [0.009-0.083] | (4.0%) |
|  | *ns* | *ns* |  | **** |  | ***** |  |
| Local | 1.13 [0.88-1.21] | 1.21 [1.09-1.42] |  | 0.005 [0.002-0.007] | (0.5%) | 0.030 [0.014-0.057] | (3.0%) |
| Systemic | 1.13 [0.92-1.42] | 1.16 [1.01-1.29] |  | 0.003 [0.001-0.012] | (0.3%) | 0.041 [0.011-0.087] | (4.1%) |
|  | *ns* | *ns* |  | *ns* |  | *ns* |  |
| CU | 1.16 [1.00-1.40] | 1.15 [1.00-1.31] |  | 0.006 [0.003-0.010] | (0.6%) | 0.030 [0.013-0.066] | (3.0%) |
| OAS | 1.15 [1.00-1.49] | 1.23 [1.08-1.43] |  | 0.004 [0.001-0.007] | (0.4%) | 0.025 [0.012-0.069] | (2.5%) |
| GI | 0.72 [0.37-1.21] | 1.16 [1.03-1.46] |  | 0.004 [0.003-0.005] | (0.4%) | 0.025 [0.012-0.049] | (2.5%) |
| U/AE | 1.13 [0.89-1.40] | 1.15 [1.02-1.25] |  | 0.003 [0.001-0.011] | (0.3%) | 0.041 [0.009-0.082] | (4.1%) |
| AN | 1.29 [1.20-138] | 1.21 [0.99-1.43] |  | 0.014 [0.007-0.021] | (1.4%) | 0.043 [0.016-0.119] | (4.3%) |
|  | *ns* | *ns* |  | *ns* |  | *ns* |  |

Pru p 3/Peach sIgE and Pru p 3/Total IgE ratio median and IQR values according to tolerance/allergenicity or avoidance and symptoms classification. GrLOW: Pru p 3 sIgE from 0.1-0.34 kU_A_/L; GrB: Pru p 3 sIgE >0.35 kU_A_/L. Local (CU: contact urticaria; OAS: oral allergy syndrome; GI: gastrointestinal symptoms), systemic symptoms (U/AE: generalized urticaria or angioedema; AN: anaphylaxis). Mann Whitney U-test and Kruskal-Wallis test were used to verify significance. Patients from the group AV (avoid) were not included on the statistical analysis because tolerance or allergy could not be guaranteed. Asterisks express significance of p value * 0.01 to 0.05, ** 0.001 to 0.01, *** 0.0001 to 0.001, **** <0.0001 and ns non-significant.

**Supplementary Table 2. Sensitization to LTPs from other allergenic sources**

|  | **Total**  **Sensitized** | | **Mal d 3** | | **Tri a 14** | | **Jug r 3** | | **Cor a 8** | | **Ara h 9** | |
| --- | --- | --- | --- | --- | --- | --- | --- | --- | --- | --- | --- | --- |
| ***GrLOW*** (n=70) | 45 | (64.3%) | 32 | (45.7%) | 5 | (7.1%) | 15 | (21.4%) | 5 | (7.1%) | 18 | (25.7%) |
| ***GrB***  (n=318) | 307 | (96.2%) | 254 | (79.9%) | 172 | (54.1%) | 258 | (81.1%) | 239 | (75.2%) | 253 | (79.6%) |

The total patients analyzed are included for GrLOW (Group Low) and GrB (Group B). From them, the total sensitized to one or more non-Pru p 3 LTPs and the percentage they represent are included. Additionally, the number and percentage of sensitized per LTP (Mal d 3, Tri a 14, Jug r 3, Cor a 8, Ara h 9) are described.

# Supplementary Figures

**Figure 2. Co-sensitization to other LTPs.**

Co-sensitization is represented as % of sensitization. OR: Odds ratio (<1 inverse or >1 direct association); 95% CI: 95% Confidence interval. Fisher’s exact test and OR (95% CI) as an association measurement were used to test statistical significance. Asterisks express significance of p value (* 0.01 to 0.05, ** 0.001 to 0.01, *** 0.0001 to 0.001, **** <0.0001 and ns non-significant).
