# Supplementary figures and images for: Low Levels Matter: Clinical Relevance of Low Pru p 3 sIgE in Patients With Peach Allergy
Source: Front Allergy. 2022 Apr 5;3:868267. doi: 10.3389/falgy.2022.868267 (PMC9234939; doi:10.3389/falgy.2022.868267)

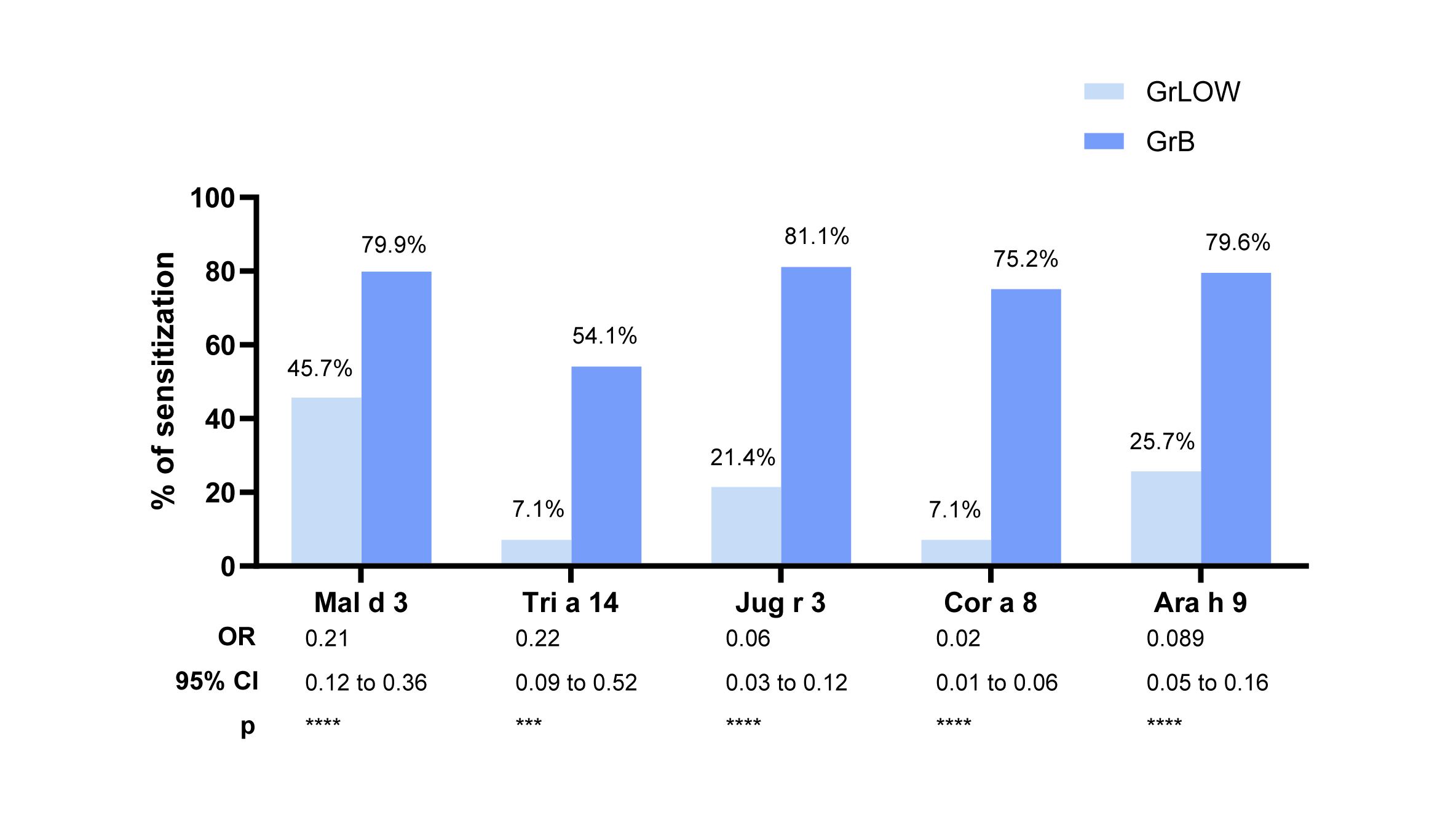

Supplement: Supplementary file 2 [file Image_1.TIF]
